# Nucleolin recognizing silica nanoparticles inhibit cell proliferation by activating the Bax/Bcl-2/caspase-3 signalling pathway to induce apoptosis in liver cancer

**DOI:** 10.3389/fphar.2023.1117052

**Published:** 2023-02-09

**Authors:** Liangliang Xiang, Yun Li, Xinyu Gu, Shujie Li, Junwei Li, Jinlong Li, Yongxiang Yi

**Affiliations:** ^1^ The Second Hospital of Nanjing, Nanjing University of Chinese Medicine, Nanjing, China; ^2^ Department of Traditional Chinese Medicine, Fujian Medical University Union Hospital, Fuzhou, Fujian, China

**Keywords:** MSNs, AS1411, hepatocellular carcinoma, proliferation, apoptosis

## Abstract

Multifunctional nanocarrier platforms have shown great potential for the diagnosis and treatment of liver cancer. Here, a novel nucleolin-responsive nanoparticle platform was constructed for the concurrent detection of nucleolin and treatment of liver cancer. The incorporation of AS1411 aptamer, icaritin (ICT) and FITC into mesoporous silica nanoparticles, labelled as Atp-MSN (ICT@FITC) NPs, was the key to offer functionalities. The specific combination of the target nucleolin and AS1411 aptamer caused AS1411 to separate from mesoporous silica nanoparticles surface, allowing FITC and ICT to be released. Subsequently, nucleolin could be detected by monitoring the fluorescence intensity. In addition, Atp-MSN (ICT@FITC) NPs can not only inhibit cell proliferation but also improve the level of ROS while activating the Bax/Bcl-2/caspase-3 signalling pathway to induce apoptosis *in vitro* and *in vivo*. Moreover, our results demonstrated that Atp-MSN (ICT@FITC) NPs had low toxicity and could induce CD3^+^ T-cell infiltration. As a result, Atp-MSN (ICT@FITC) NPs may provide a reliable and secure platform for the simultaneous identification and treatment of liver cancer.

## 1 Introduction

Primary liver cancer is the sixth most prevalent malignancy and the second main cause of death from cancer worldwide (Li et al., 2021a; Llovet et al., 2022). The high death rate of liver cancer has generated global concern. Limited therapeutic options exist for hepatocellular carcinoma (HCC) patients, and current management strategies mainly consist of surgical resection, chemotherapy and biotherapy (Yang and Heimbach, 2020). Chemotherapy can help patients live longer, but the indiscriminate attack of chemical medicines on both normal and malignant cells may cause severe side effects. Therefore, it is necessary to build a targeted drug delivery system to improve the bioavailability of non-targeted payloads and reduce the potential side effects on normal cells.

Fortunately, targeted nanoplatforms have been recently developed to address these issues. Several intracellular research studies have confirmed that nanomaterials have a lot of promise to construct drug delivery platforms (Dad et al., 2021; Zhao et al., 2021; Costoya et al., 2022). In particular, accumulating evidence has suggested that mesoporous silica nanoparticles (MSNs) play a vital role in drug delivery systems due to their low toxicity, superior biocompatibility, a large pore volume and surface area, and simplicity of functionalization (Chen et al., 2022; Wei et al., 2022). Thus, MSNs have been widely utilized to load DNA (Lin-Shiao et al., 2022; Ma et al., 2022), drugs (Song et al., 2022; Zhang et al., 2022) and light (Semcheddine et al., 2021; Jena et al., 2022) for cancer therapy and diagnosis. Furthermore, in order to increase the targeting efficiency, nanoparticles modified with a DNA or RNA aptamer are considered to be more promising owing to their low immunogenicity, smaller size, and active targeted property. Aptamers hold great promise in cancer detection (Wu et al., 2021a; Yu et al., 2021a) and therapy (Ouyang et al., 2020; Zhang et al., 2020), especially in optimizing targeted drug delivery (Chen et al., 2016; Porciani et al., 2018). Notably, their high specificity, strong affinity, and low cost have been intensively debated. The nucleolin aptamer AS1411 is a short oligonucleotide targeting agent that binds to nucleolar proteins (Wu et al., 2022; Zhao et al., 2022). Nucleolin is overexpressed on the membrane of liver cancer cells (Lai et al., 2014). Previous studies have suggested that AS1411-modified nanoparticles (NPs) could be used as cancer-targeting drug carriers (Yang et al., 2018; He et al., 2020). Therefore, it is of interests to combine the properties of AS1411 and MSNs for targeted cancer therapy via the specific AS1411−nucleolin interaction.

On the other hand, recently, experimental evidence has shown that icaritin (ICT) can be an effective anticancer agent against liver cancer. A clinical study found that patients with advanced liver cancer benefited from ICT therapy (Qin et al., 2020), which promoted apoptosis in HCC by inhibiting alpha-fetoprotein (AFP) expression (Li et al., 2021b). Regretfully, the insolubility and short stay in the body severely limit the clinical applications of ICT. Thus, the construction of MSNs incorporating ICT and AS1411 may not only resolve the issues of treatment, but also show the ability to identify. Nevertheless, few studies have successfully combined these properties together to achieve an efficient cancer identification and treatment. (Tarannum et al., 2022).

Herein, a nanoplatform based on MSNs was applied to achieve integrated diagnosis and therapy simultaneously in HCC. (Joseph et al., 2020). In this nanoplatform, AS1411 can bind to nucleolin on the cell membrane surface as a biogate. In the absence of nucleolin, the nanoplatform exists in a closed state due to the quenching effect of the immobilized black hole quencher-1 (BHQ-1) on FITC and the blocking voids on the surface of the MSNs by AS1411. When the nanoplatform encounters nucleolin, (Liu et al., 2020), AS1411 specifically binds to nucleolin, resulting in the release of FITC and ICT from the MSNs to achieve the goal of combined diagnosis and treatment. The synthesis and size of the nanoplatform were characterized by utilizing transmission electron microscopy (TEM), a microplate reader and dynamic light scattering (DLS). The tracellular fluorescence released by the Atp-MSN (ICT@FITC) NPs in the human hepatoma cell line HCCLM3 and L02 was measured with a microplate reader and flow cytometry. Moreover, the cytotoxicity of the Atp-MSN (ICT@FITC) NPs to HCCLM3 and HepG2 cells and their effects on proliferation and apoptosis were studied by the colony formation, Annexin V-FITC/PI kit, western blotting *etc.* Finally, the therapeutic efficacy and toxicity were investigated in C57BL/6 mice. This work provides a new nanoplatform for the simultaneous identification and treatment of liver cancer.

## 2 Materials and methods

### 2.1 Reagents

Tetraethyl orthosilicate (TEOS), n-cetyltrimethylammonium bromide (CTABr) (3-aminopropyl) triethoxysilane (APTES), N-hydroxysulfosuccinimide (NHS), BHQ-1 and 1-(3-dimethylaminopropyl)-3-ethylcarbodiimide hydrochloride (EDC) were all purchased from Sigma‒Aldrich. The nucleolin aptamer (5-GGT​GGT​GGT​GGT​TGT​GGT​GGT​GGT​GG-3) was synthesized by Sangon Biotech.

### 2.2 Methods

#### 2.2.1 Synthesis of mesoporous silica nanoparticles (MSNs) and Atp-MSN (ICT@FITC) NPs

MSNs were synthesized according to a previously described method (Qian et al., 2013). Specifically, CTABr (0.052 g) was dissolved in 25 mL of deionized water, and 1 mL of NaOH solution (0.36 M) was added. A condensation reflux apparatus was utilized to heat the aforementioned liquid to 95°C while stirring continuously. 2 mL of TEOS was then added dropwise to the hot mixed solution, followed by stirring at 95°C for 3 h. After filtration and washing with deionized water and ethanol, the white precipitate was carefully dried in an oven at 60°C and then heated in a muffle furnace at 550°C for 5 h to remove the residual templating agent. For Atp-MSN (ICT@FITC) synthesis, 1 mL of APTES was added to a suspension of 1.0 g of MSNs and 100 mL of absolute ethanol. To obtain a solid white APTES-MSN powder, the complex was continuously stirred at 36°C for 6 h, filtered, washed with ethanol, and dried at 60°C. 1 mL of deionized water containing 10 mg of APTES-MSNs, 1 mg of EDC, 2.5 mg of NHS and 0.1 mg of BHQ-1 was stirred at room temperature for 4 h. After washing, filtration, and resuspension, 0.5 mg of ICT and 0.5 mg of FITC were added to the mixture, which was shaken at room temperature overnight. After filtering, MSN (ICT@FITC) was obtained. Finally, the samples were centrifuged, washed, and dried at room temperature. 1 mL of buffer (10 mM Tri-HCl, 1 mM EDTA, 50 mM NaCl, 10 mM MgCl_2_) containing 1 mg of MSN (ICT@FITC) and 10 μL of AS1411 (100 μM) was shaken at 37°C for 1 h, followed by centrifugation, washing and resuspension for storage at 4°C.

#### 2.2.2 Encapsulation efficiency (EE) and loading efficiency (LE)

The amount of ICT/FITC in Atp-MSN (ICT@FITC) NPs was analyzed by a spectroscopic method. PBS was serially diluted to obtain solutions with concentrations of 25, 50, 100, 200, 300, 400, and 500 mg/mL. UV spectrophotometer at 380 nm and 460 nm was performed to measure the infrared absorption of ICT and FITC, and the calibration curve of absorbance (Y) and concentration (X) was constructed by linear regression. The entrapment efficiency and loading efficiency were calculated using the following formula: entrapment efficiency = final loaded ICT/FITC/initial feed ICT/FITC × 100%. Loading efficiency = final loaded ICT/FITC/total quality (drug and nanocarrier) × 100%.

#### 2.2.3 Flow cytometric analysis

At least 10,000 viable were detected by flow cytometry after incubating 5×10^5^ HCCLM3 and L02 cells with the nanocomplexes for various times at different concentrations (Yu et al., 2020).

#### 2.2.4 Cell culture

The human hepatocarcinoma cell lines HCCLM3 and HepG2 (Shanghai Institute of Biochemistry and Cell Biology) were cultured in high-glucose Dulbecco’s Modified Eagle’s medium (DMEM; Gibco) with 10% foetal bovine serum (FBS; HyClone) and 1% penicillin and streptomycin (HyClone). The culture conditions were 5% CO_2_ at 37°C.

#### 2.2.5 MTT assay

First, cells were seeded in 96-well plates at a density of 5×10^3^ per well. Different treatments were performed after 24 h. Next, we added MTT reagent (Alfa Aesar) to the cells and for incubation at 37°C for 4 h before removing the supernatant. The generated formazan was resuspended in 150 μL of dimethyl sulfoxide (DMSO) for absorbance measurements with a spectrometer (Hidex Chameleon) at 24, 48 and 72 h, respectively.

#### 2.2.6 Colony formation and soft agar assays

In the colony formation assay, 2×10^3^ cells seeded in a 6-well plate were treated differently and cultured for 2 weeks. The solution was updated every 2 days. Colonies were then stained with 0.2% crystal violet. In the soft agar assay, 1.2% agarose-containing DMEM was fixed at the bottom of a 6-well plate, and then 2×10^3^ cells mixed with 0.7% agarose-containing DMEM were seeded with different treatments. After 2 weeks of culture and the solution was updated every 2 days, colonies were stained with 0.2% crystal violet.

#### 2.2.7 ROS production

HCCLM3 and HepG2 cells (5×10^4^ cells/mL) were treated differently for 6 h and then washed three times with PBS. The cells in each well were then treated with the reagents in the intracellular oxidative stress ROS primary green fluorescence detection kit (Sigma‒Aldrich). Then, fluorescence images of the cells were acquired using a fluorescence microscope.

#### 2.2.8 Apoptosis assay

To detect apoptosis, an Annexin V-FITC/PI kit (BD Biosciences) was used. After different treatments, cells in a six-well plate were washed twice with PBS before the addition of Annexin V and the propidium iodide (PI) reagent for 30 min in the dark at room temperature. Nuclei were counterstained with DAPI (Thermo Fisher Scientific).

#### 2.2.9 Western blot

Briefly, all proteins, including phosphatase and protease inhibitors, were extracted from cells using RIPA buffer, and protein concentrations were determined using a BCA kit. SDS‒PAGE was used to separate proteins, which were transferred to PVDF membranes. Membranes were blocked with 5% skim milk for 2 h and then treated with the primary antibody overnight and the rabbit secondary antibody for 1.5 h. Protein bands were detected by enhanced chemiluminescence (ECL; Bio-Rad Laboratories). The anti-Bax, anti-Bcl-2, anti-caspase-3, anti-cleaved caspase-3 and rabbit secondary antibodies were obtained from Abcam (Zeng et al., 2022).

#### 2.2.10 Xenograft mouse model

All animal studies were approved by the Institutional Animal Care and Use Committee of the Second Hospital of Nanjing. Twelve 6-week-old female C57BL/6 mice from the Nanjing University of Chinese Medicine were divided into three groups according to treatment: PBS, MSNs and Atp-MSN (ICT@FITC) NPs. A total of 1×10^7^ HCCLM3 cells in 200 µL of PBS were implanted subcutaneously into the mice. The mice were divided into three groups and given PBS, MSNs (10 mg/kg), or Atp-MSN (ICT@FITC) NP (10 mg/kg) every 3 days for a total of six injections *via* the tail vein. Three days later, the mice were sacrificed for immunohistochemical, apoptosis, immune effects and toxicity analyses and evaluations of the blood, organs and tumours.

#### 2.2.11 Immunohistochemistry (IHC)

The immunostaining method was performed following a previous study (Qin et al., 2022). After tumour tissues were embedded and sliced, they were washed with xylene and absolute ethyl alcohol 3 times. Sodium citrate (10 mmol/L) was utilized for the antigen retrieval step, and the samples were incubated at 95°C for 30 min. Finally, serum blocking was performed for 30 min. The anti-Bax, anti-Bcl-2, anti-caspase-3, anti-cleaved caspase-3, anti-Ki67 and rabbit secondary antibodies were obtained from Abcam.

#### 2.2.12 Immunofluorescence staining

After fixation, infiltration and blocking with 1% bovine serum albumin (BSA), the tumours were incubated with antibodies overnight at 4°C. DAPI was used for nuclear staining. The sections were observed using confocal microscopy (Zeiss LSM 710).

#### 2.2.13 Statistical analysis

GraphPad Prism 8 (GraphPad Software) was used for statistical analysis. Data between two groups were analyzed by independent Student’s t-test. All data were presented as mean ± SD. ^★^ *p* < 0.05, ^★★^ *p* < 0.01, ^★★★^ *p* < 0.001.

## 3 Results and discussion

### 3.1 Synthesis and characterization of the MSNs and Atp-MSN (ICT@FITC) NPs

**SCHEME 1:**
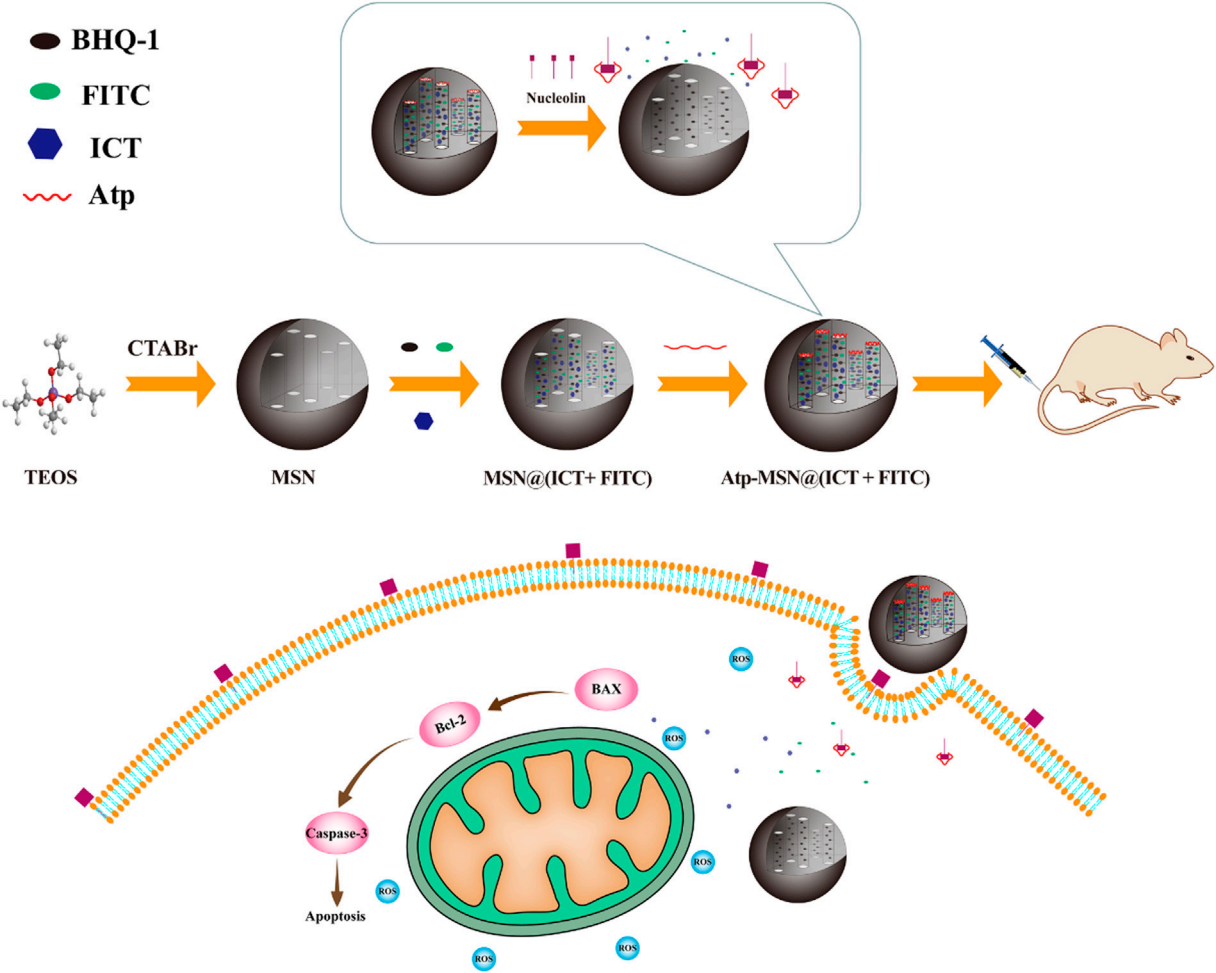
Schematic of the synthesis and antitumor mechanism of Atp-MSN (ICT@FITC) NPs against liver cancer.

**FIGURE 1:**
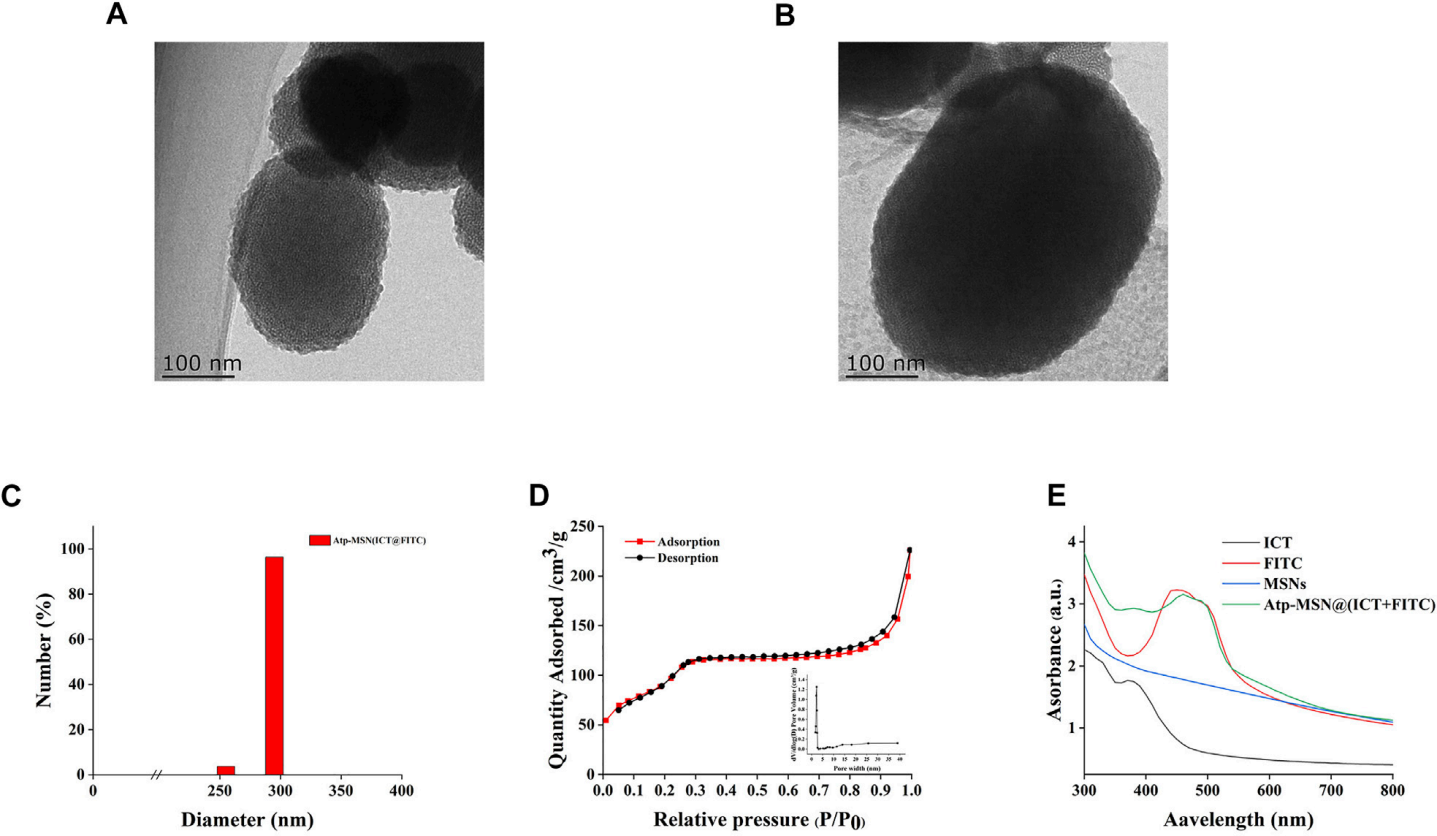
Synthesis and characterization of Atp-MSN (ICT@FITC) NPs **(A)** TEM images of MSNs (scale = 100 nm) **(B)** Atp-MSN (ICT@FITC) NPs (scale = 100 nm) **(C)** Hydrated particle size of the Atp-MSN (ICT@FITC) NPs **(D)** BET isotherm and BJH pore size distribution (inset) of Atp-MSN (ICT@FITC) NPs **(E)** Ultraviolet-visible (UV) absorption spectra of ICT, FITC, MSNs, and Atp-MSN (ICT@FITC) NPs, respectively.

The synthesis of MSNs, Atp-MSN (ICT@FITC) NPs and the antitumor mechanism are depicted in Scheme 1. Transmission electron microscopy (TEM) was used to observe the morphologies of MSNs and Atp-MSN (ICT@FITC) NPs. The results clearly showed that they were both spherical (Figures 1A, B). The mean hydrodynamic diameter of the Atp-MSN (ICT@FITC) NPs measured by DLS was 295 nm (Figure 1C). An adsorption investigation of Atp-MSN (ICT@FITC) NPs at P/P_0_ value showed a conventional Type IV isotherm, demonstrating the presence of typical meso-scale pores. The specific surface calculated by the BET model was 329.14 m^2^/g and the pore distance was determined to be 4.44 nm (Figure 1D). To accurately evaluate the drug encapsulation, ultraviolet‒visible (UV‒vis) spectrophotometry was carried out. The maximum absorption peaks of Atp-MSN (ICT@FITC) NPs appeared at approximately 380 and 460 nm (Figure 1E), corresponding to the characteristic peaks of ICT and FITC. This result revealed that both ICT and FITC were successfully encapsulated. In addition, a linear relationship between OD value and the concentration of ICT and FITC was established. The calibration curves were Y = 0.0818x + 0.1752 (ICT) and Y = 0.0545x + 0.3167 (FITC). The encapsulation efficiency of ICT was 50.32% ± 3.08%, and loading quantity was 6.32% ± 1.23%. The encapsulation efficiency of FITC was 42.57% ± 3.23%, and loading quantity was 5.35% ± 1.05%.

### 3.2 Performance of the Atp-MSN (ICT@FITC) NPs

**FIGURE 2:**
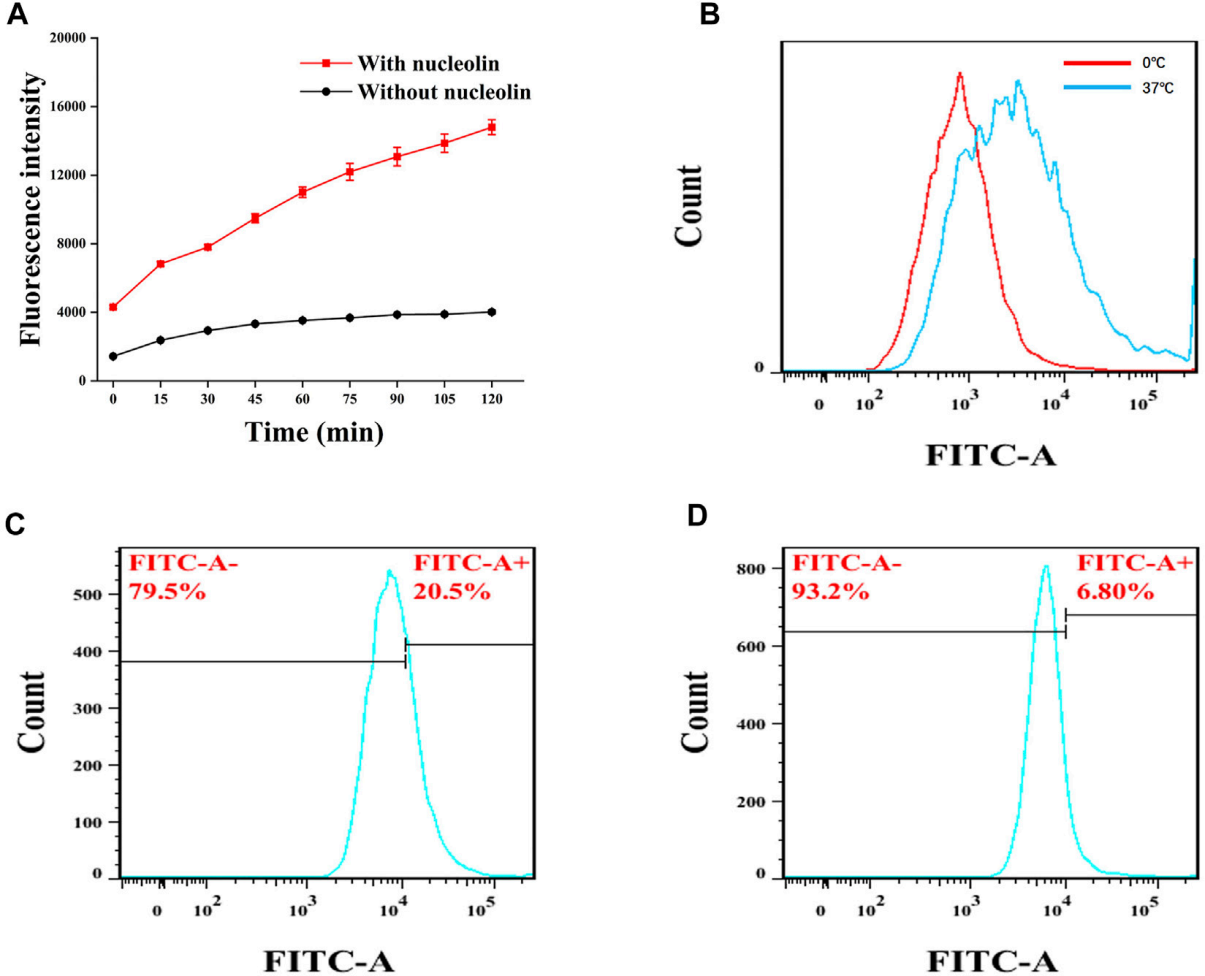
FITC release from Atp-MSN (ICT@FITC) NPs and nucleolin detection **(A)** Detection of nucleolin **(B)** FITC was release from Atp-MSN (ICT@FITC) NPs at different temperatures (0°C and 37°C). Cellular uptake of Atp-MSN (ICT@FITC) NPs by HCCLM3 **(C)** and L02 **(D)** cells.

The ability of Atp-MSN (ICT@FITC) NPs to detect nucleolin was evaluated *in vitro* experiments. As shown in Figure 2A, the fluorescence intensity in the presence of nucleolin was significantly stronger than that in the absence of nucleolin, implying that Atp-MSN (ICT@FITC) NPs could detect the nucleolin. This result was consistent with previous study that AS1411 modified nanocomplexes showed a targeting capability to nucleolin (Charbgoo et al., 2021). Additionally, it was found that high temperature (37°C) was more favourable for the nanoplatform release than low temperature (4°C, Figure 2B). Flow cytometry analysis showed that the fluorescence intensity in the HCCLM3 cells (Figure 2C) was stronger than that of the L02 cells (Figure 2D), suggesting that Atp-MSN (ICT@FITC) NPs could accumulate in liver cancer cells. In addition, the experimental conditions were optimal at a dose of 20 µL (1 mg/mL) (Supplementary Figure S1A) and a time of 1.5 h (Supplementary Figure S1B). Selectivity experiments revealed that Atp-MSN (ICT@FITC) NPs were more selective for nucleolin than Bovine Serum Albumin (BSA), Matrix Matalloproteinase 2 (MMP-2), Matrix Matalloproteinase 9 (MMP-9) and *α*-fetoprotein (AFP) (Supplementary Figure S2).

### 3.3 Cell proliferation and apoptosis analysis

**FIGURE 3:**
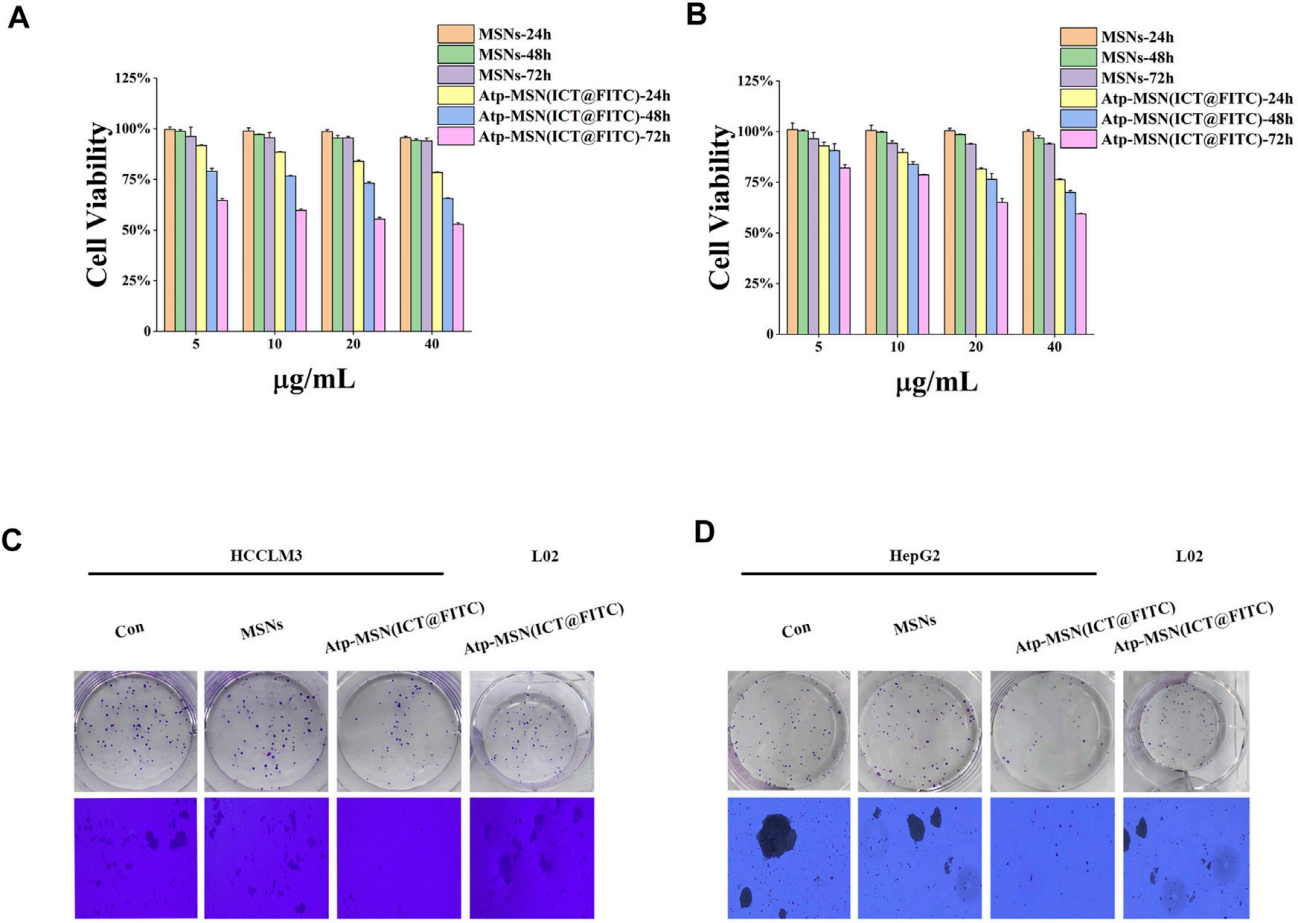
Atp-MSN (ICT@FITC) NPs inhibit HCC cell proliferation. Cytotoxicity studies in HCCLM3 **(A)** and HepG2 **(B)** HCC cells treated with different concentrations of MSNs and Atp-MSN (ICT@FITC) NPs for different lengths of time (*n* = 3). Colony formation **(C)** and soft agar colony formation assays **(D)** with HCCLM3, HepG2 and L02 cells treated with MSNs and Atp-MSN (ICT@FITC) NPs.

**FIGURE 4:**
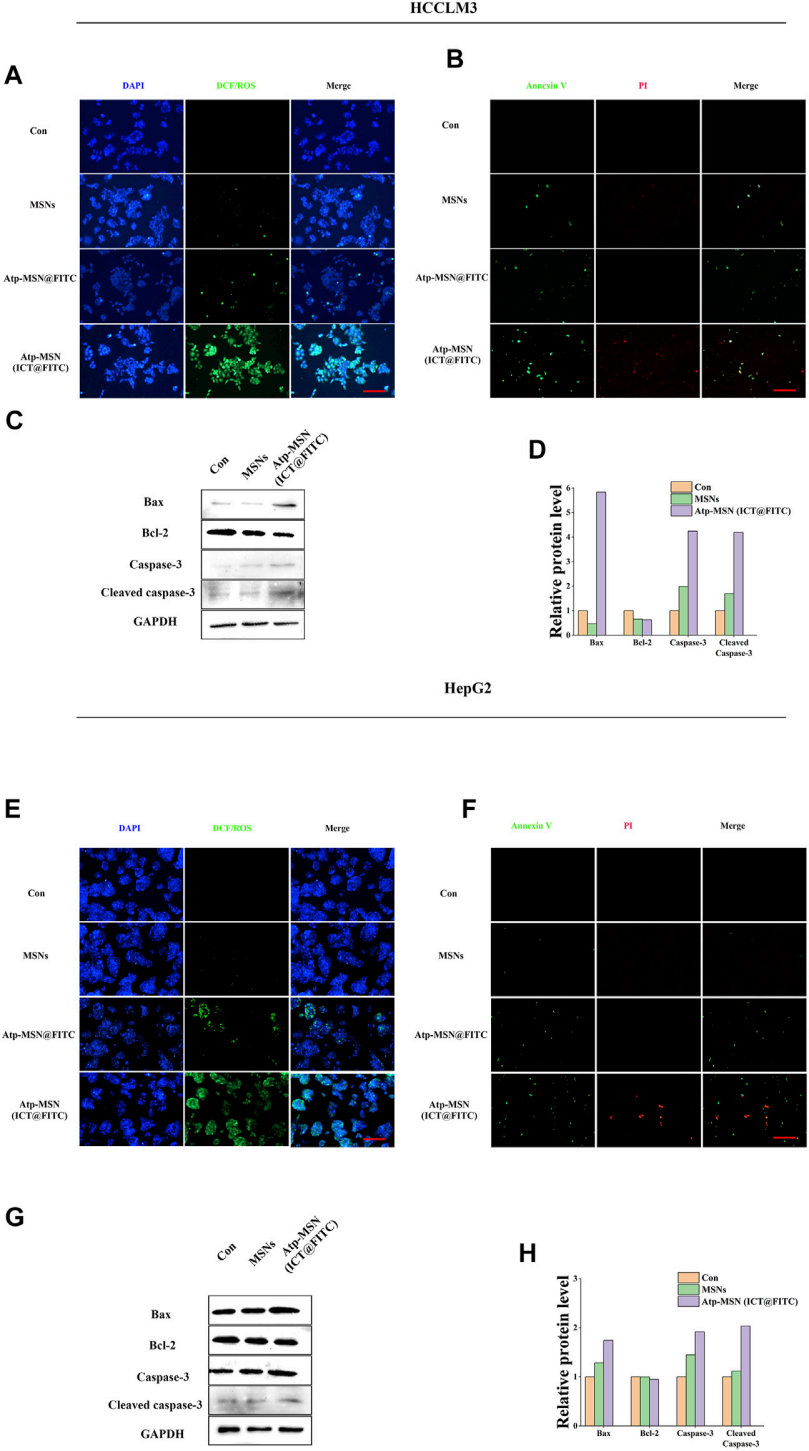
Atp-MSN (ICT@FITC) NPs effect on the apoptosis in tumor cells **(A)** and **(E)**, Reactive oxygen species (ROS) produced after treatment with MSNs and Atp-MSN (ICT@FITC) NPs. DAPI (blue); DCF/ROS (green) **(B)** and **(F)**, Annexin V-FITC and PI triple fluorescent staining showed cellular apoptosis after treatment with MSNs and Atp-MSN (ICT@FITC) NPs. DAPI: blue; Annexin V: green; PI: red (scale = 100 nm) **(C)** and **(G)**, Immunoblotting for Bax, Bcl-2, caspase-3 and cleaved caspase-3 (normalized to GAPDH) **(D)** and **(H)**, Quantification of Bax, Bcl-2, caspase-3 and cleaved caspase-3.

Previous studies have confirmed that ICT is directly toxic to a variety of cancer cells (Wang et al., 2015; Guo et al., 2022). To confirm the antitumor effect of Atp-MSN (ICT@FITC) NPs against HCC, MTT assays were conducted to determine the viability of HCCLM3 and HepG2 HCC cells. We found that Atp-MSN (ICT@FITC) NPs reduced the viability of both cells in dose- and time-dependent manner (Figures 3A, B). When the concentration of Atp-MSN (ICT@FITC) NPs increased to 20 μg/ml in 48 h, the viability of HCCLM3 and HepG2 cells decreased to approximately 75%. Therefore, 20 μg/ml in 48 h was employed in the following experiments. After treatments with Atp-MSN (ICT@FITC) NPs, colony formation and soft agar assays were performed (Figures 3C, D). It was found that the proliferation of both HCCLM3 and HepG2 cells was suppressed instead of L02 cells. The ROS assay kit and Annexin V- FITC/PI kit were used to determine whether there was a link between Atp-MSN (ICT@FITC) NPs and apoptosis. Compared with the Con, MSNs and ATP-MSN@FITC groups, the Atp-MSN (ICT@FITC) group showed the strongest fluorescence signals of ROS, Annexin V and PI. These results revealed that Atp-MSN (ICT@FITC) NPs could increase the level of cellular ROS (Figures 4A, E) and induce cell apoptosis (Figures 4B, F).

Western blotting was performed to explore the apoptosis mechanism of Atp-MSN (ICT@FITC) NPs. The expression levels of Bax, caspase-3 and cleaved caspase-3 were upregulated, while that of Bcl-2 was downregulated after treatments with Atp-MSN (ICT@FITC) NP. The outcomes implied that Atp-MSN (ICT@FITC) NPs could activate the Bax/Bcl-2/caspase-3 signalling pathway to induce apoptosis in HCC cells (Figures 4C, G). The quantification of Bax, Bcl-2, caspase-3 and cleaved caspase-3 were shown in (Figures 4D, H), which supported the conclusion.

### 3.4 *In Vivo* analysis of Atp-MSN (ICT@FITC) NPs to tumour growth and apoptosis

**FIGURE 5:**
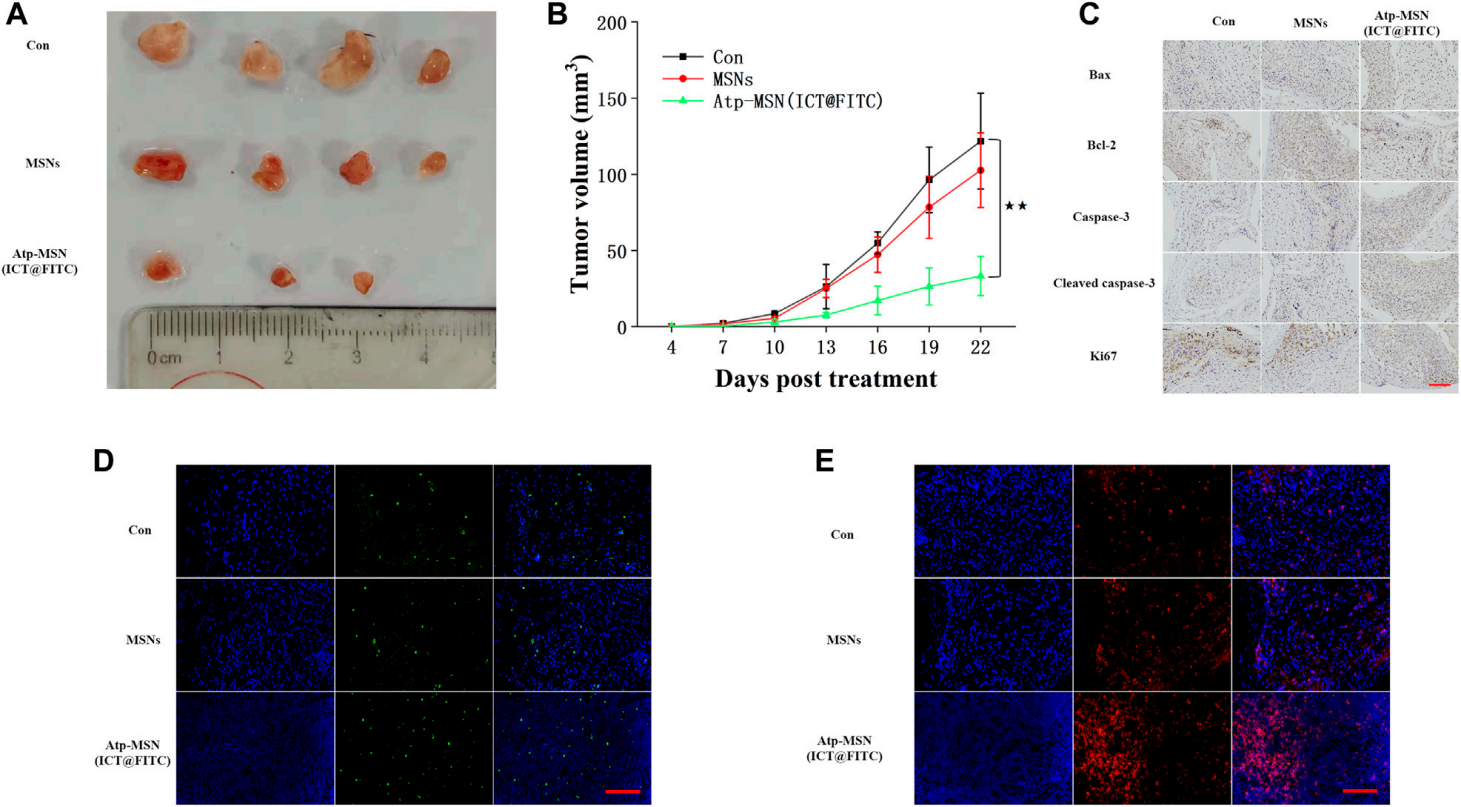
Atp-MSN (ICT@FITC) NPs inhibited HCC cell proliferation and induced apoptosis *in vivo* **(A)** Physical photographs of tumours from the Con, MSN and Atp-MSN (ICT@FITC) NP groups **(B)** Tumor growth curve of HCCLM3-bearing mice in three groups (n = 4), *p* < 0.01 **(C)** Evaluation of Bax, Bcl-2, caspase-3, cleaved caspase-3 and Ki67 protein expression by IHC **(D)** The average number of apoptotic cells detected by TUNEL immunofluorescence staining **(E)** Fluorescence image monitoring of immune cell infiltration (scale = 100 nm).

To evaluate the antitumor effects of Atp-MSN (ICT@FITC) NPs *in vivo*, HCCLM3 xenograft models were constructed. Three days after subcutaneous inoculation, mice were injected via the tail vein every 3 days for a total of four injections. Mice were treated with PBS, MSNs (10 mg/kg), and Atp-MSN (ICT@FITC) NPs (10 mg/kg), respectively. We found that the nanoparticles exhibited the most powerful therapeutic effect to reduce the tumour volume (Figures 5A, B). Next, we assessed the expression of Bax, Bcl-2, caspase-3, cleaved caspase-3 and Ki67 by IHC. These results were the same as those obtained from the *in vitro* experiments (Figure 5B). TUNEL analysis suggested that apoptotic cells were significantly increased after Atp-MSN (ICT@FITC) NPs treatments (Figure 5C). CD^3+^ T-cell infiltration mainly occurred in the Atp-MSN (ICT@FITC) NP group (Figure 5D), which indicated that Atp-MSN (ICT@FITC) NPs activated non-specific immunity. Thus, Atp-MSN (ICT@FITC) NPs inhibited tumour growth and induced apoptosis *in vivo*.

**FIGURE 6:**
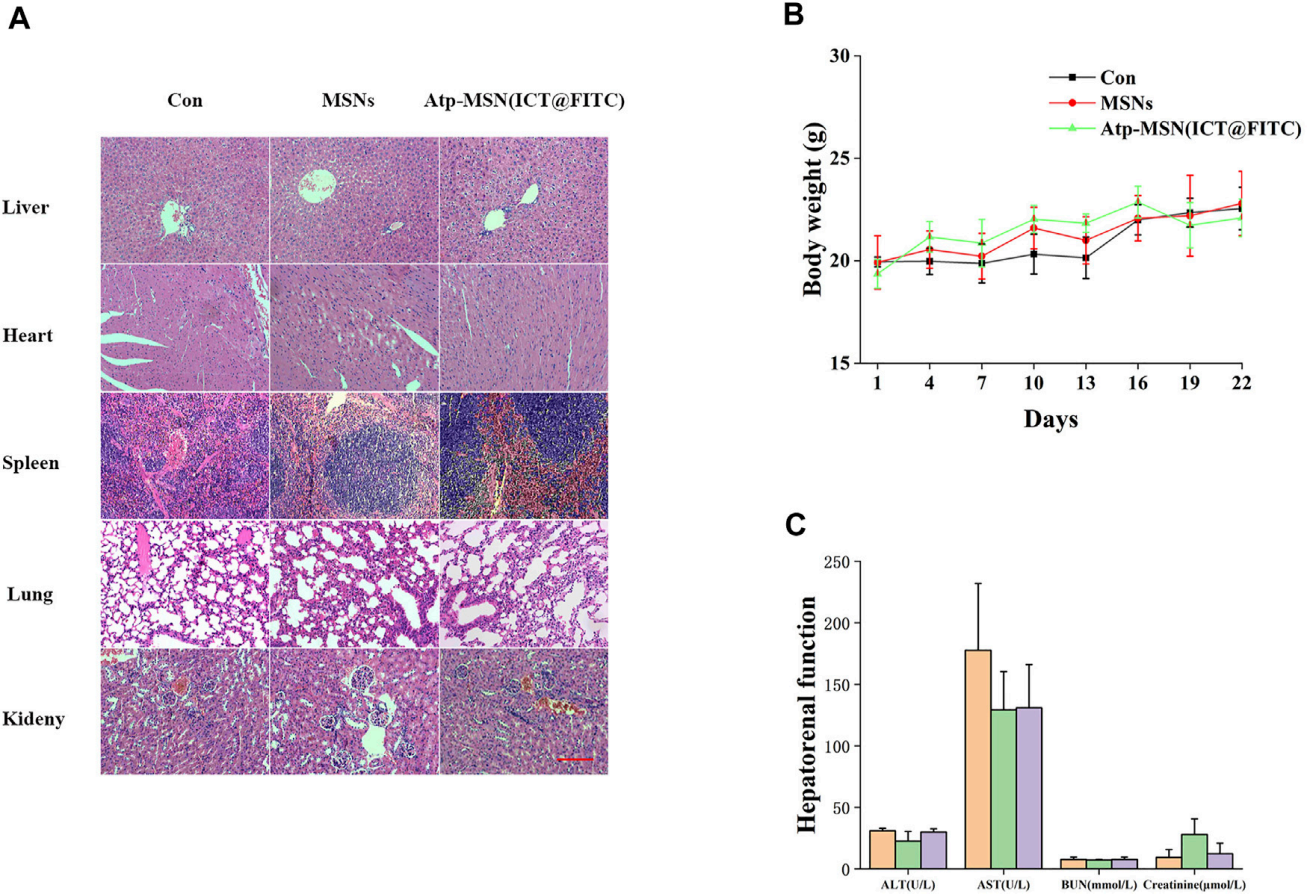
**(A)** H&E staining of the major organs from mice treated with different therapies (scale = 100 nm) **(B)** Mouse body weight changes (n = 4) **(C)** Mice hepatorenal function test (*n* = 4).

To further assess the safety of Atp-MSN (ICT@FITC) NPs, we performed biosafety-related serological toxicological and pathological analyses. Pathological staining for haematoxylin-eosin (H&E) did not show severe damage to the mouse organs, including the heart, lung, liver, spleen and kidney (Figure 6A). During the experiment, the body weights of the mice remained stable (Figure 6B), and the liver and kidney functions in each group were within the normal range (Figure 6C). Therefore, Atp-MSN (ICT@FITC) NPs showed low toxicity.

## 4 Discussion

HCC, one of the most widespread cancers worldwide, remains a major global health problem (Missiaen et al., 2022). Due to the complex environment in the body, natural products cannot be effectively delivered to the liver tumor site, which also limits their application. Drug delivery systems mediated by MSNs have demonstrated significant benefits in the targeted therapy, and more crucially, the logical design of MSNs allows for the simultaneous integration of numerous functionalities into a single platform for drug delivery (Vallet-Regí et al., 2022; Yin et al., 2022), diagnosis (Wang et al., 2021; Tang et al., 2022) and even combination therapy (Wu et al., 2021b; Li et al., 2021c). For example, dual drug release from red light-triggered self-destructive MSNs boosted immunogenic cell death and strengthened antitumor immunity responses (Yang et al., 2022). Additionally, a novel MSN-based nanoplatform was designed for magnetic resonance imaging (MRI)/NIR-II fluorescence (FL) imaging and chemodynamic therapy (CDT) by adjusting the ratio of intratumoural hydrogen peroxide/glutathione (Zheng et al., 2021). Fluorescence images of FEN1 were detected in living cells based on the controlled release of the fluorescent probe from MSNs (Tang et al., 2022).

In this experiment, we evaluated the targeting ability in HCC cells *in vitro*, Atp-MSN (ICT@FITC) NPs were incubated with HCCLM3 and L02 cells, respectively. Flow cytometry results showed that the fluorescence intensity in HCCLM3 cells was significantly stronger than that in L02 cells, which indicated Atp-MSN (ICT@FITC) NPs were more likely to enter liver cancer cells. Therefore, Atp-MSN (ICT@FITC) NPs could target HCC cells (Yu et al., 2021b).

To evaluate the ability of Atp-MSN (ICT@FITC) NPs in diagnosis and treatment. Our research found the fluorescence intensity is stronger in the presence of nucleolin than in the absence of nucleolin. Compared with BSA, MMP-2, MMP-9, AFP, Atp-MSN (ICT@FITC) NPs are most sensitive to nucleolin. In addition, Atp-MSN (ICT@FITC) NPs could also inhibit the proliferation and induce apoptosis of hepatocellular carcinoma *in vitro* and *in vivo*. Atp-MSN (ICT@FITC) NPs could also activate the Bax/Bcl-2/caspase-3 signalling pathway to stimulate apoptosis *in vitro* and *in vivo*. The results of Western blot showed that Atp-MSN (ICT@FITC) NPs could increase the expression of Bax, Caspase-3 and Cleaved Caspase-3, but inhibit the expression of Bcl-2. Thus, we thought that Atp-MSN (ICT@FITC) NPs could inhibit proliferation through activating Bax/Bcl-2/caspase-3 signaling pathway to induce apoptosis *in vitro* and *in vivo*.

In our study, Atp-MSN (ICT@FITC) NPs were able to enhance CD3^+^ T-cell infiltration, so we speculated that the antitumor effect of Atp-MSN (ICT@FITC) NPs might be related to immunity. The toxicity of Atp-MSN (ICT@FITC) NPs was also evaluated. H&E staining results showed that MSNs and Atp-MSN (ICT@FITC) NPs had no obvious damage to the liver, heart, spleen, lung and kidney of mice. There was no significant change in the body weight of the mice in the Con, MSNs and Atp-MSN (ICT@FITC) NPs groups. ALT, AST, BUN and Creatinine were clinically used to evaluate liver and kidney function. Compared with the Con group, there was no significant change in liver and kidney function in the Atp-MSN (ICT@FITC) NPs group. These results all indicate that Atp-MSN (ICT@FITC) NPs have low toxicity. This method provided evidence for the safety of Atp-MSN (ICT@FITC) NPs in clinical drug development.

## 5 Conclusion

In summary, a novel nucleolin-responsive silica nanoparticle was fabricated for the detection of nucleolin and the treatment of liver cancer. The results indicated that the Atp-MSN (ICT@FITC) NP platform could not only detect nucleolin but also inhibit cell proliferation by activating the Bax/Bcl-2/caspase-3 signalling pathway, thereby inducing liver cancer cell apoptosis *in vitro* and *in vivo*. In addition, the low toxicity and activation of immunity by the nanodrug delivery system were more favourable for clinical application. Our work provides a facile strategy for constructing multifunctional nanoplatforms for cancer detection and therapy.

## Data Availability

The original contributions presented in the study are included in the article/Supplementary Materials, further inquiries can be directed to the corresponding authors.
