# Supplementary figures and images for: Nucleolin recognizing silica nanoparticles inhibit cell proliferation by activating the Bax/Bcl-2/caspase-3 signalling pathway to induce apoptosis in liver cancer
Source: Front Pharmacol. 2023 Feb 9;14:1117052. doi: 10.3389/fphar.2023.1117052 (PMC9947157; doi:10.3389/fphar.2023.1117052)

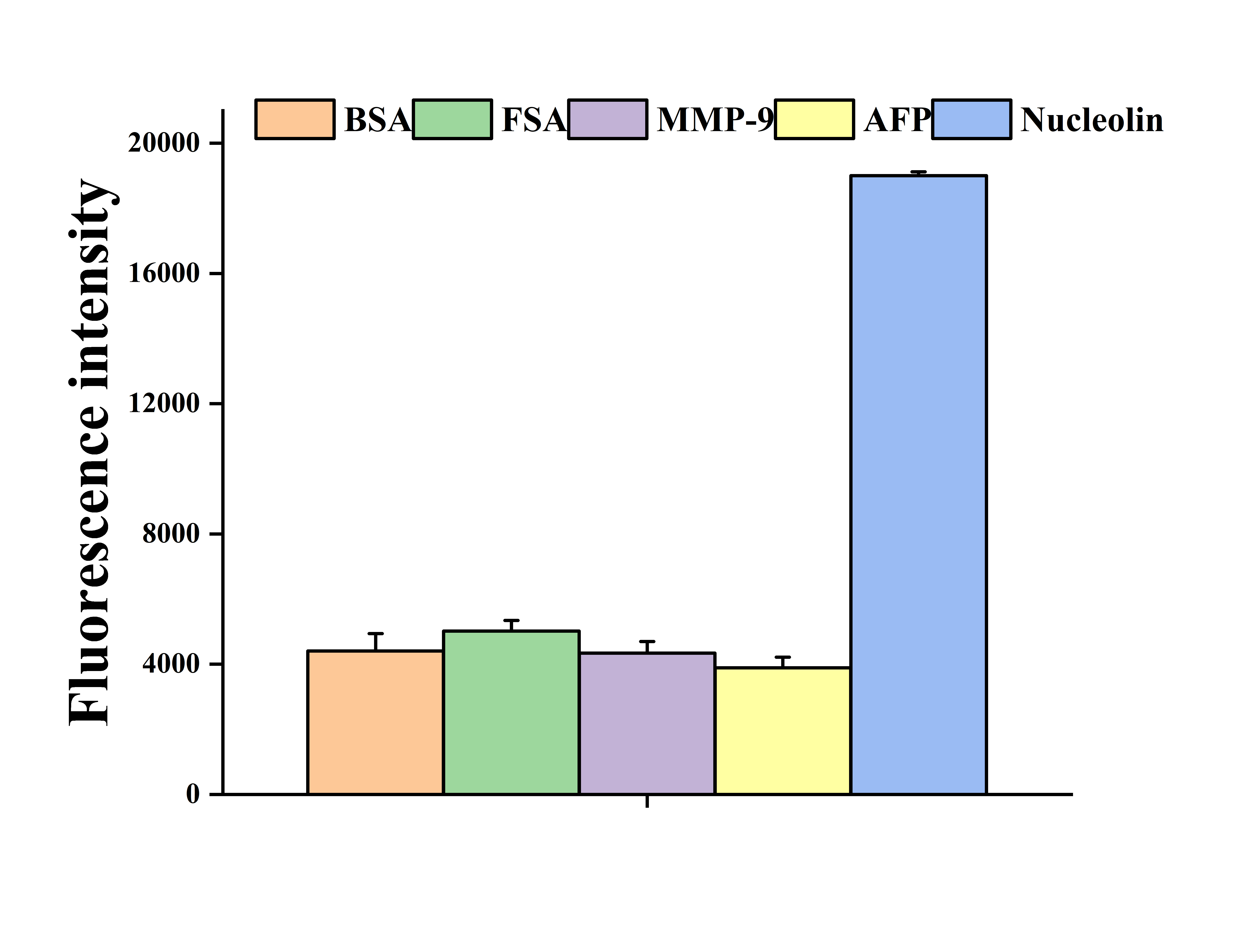

Supplement: Supplementary file 1 [file Image2.TIF]

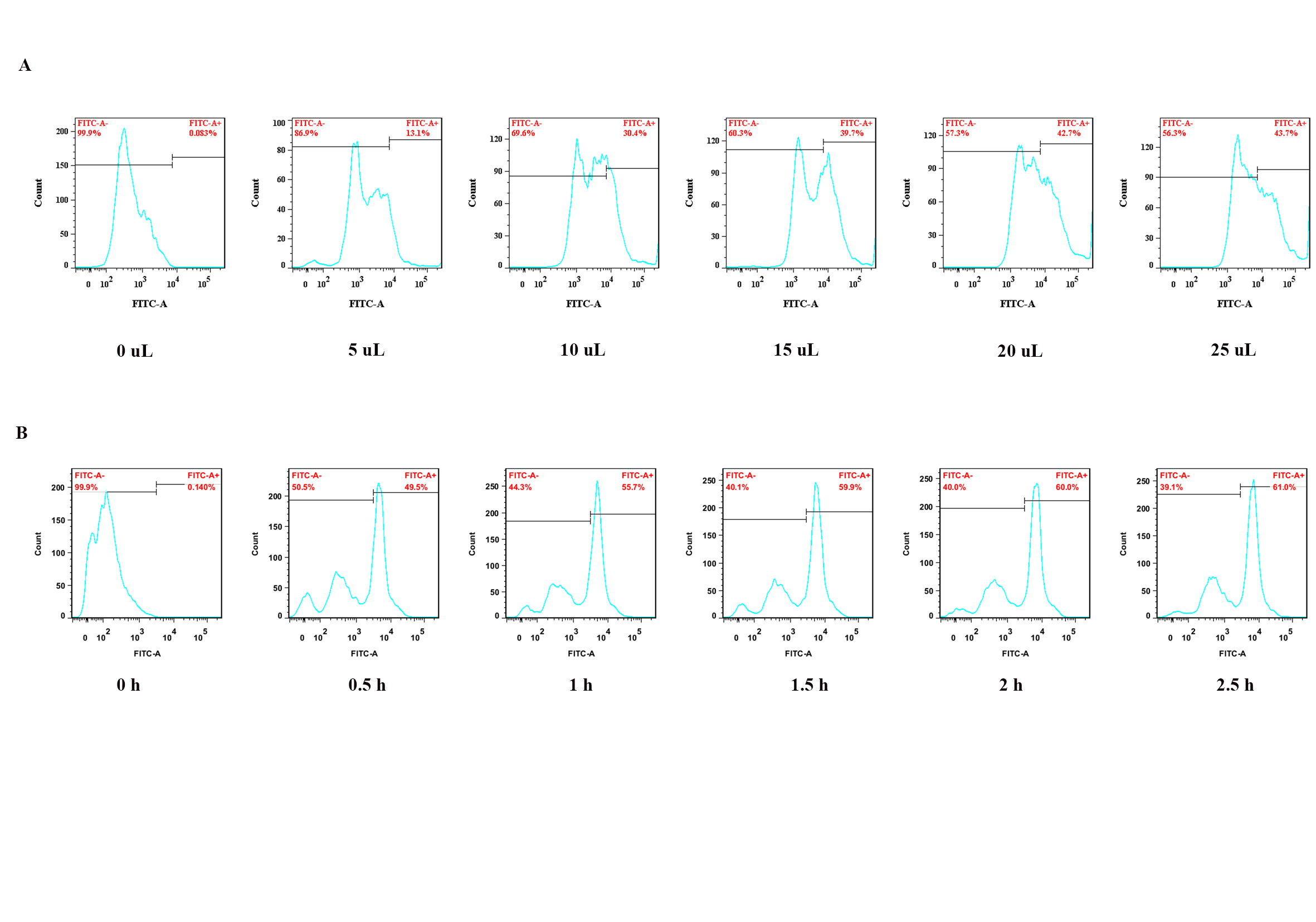

Supplement: Supplementary file 2 [file Image1.TIF]
