# Supplementary material for: Nucleolin recognizing silica nanoparticles inhibit cell proliferation by activating the Bax/Bcl-2/caspase-3 signalling pathway to induce apoptosis in liver cancer
Source: Front Pharmacol. 2023 Feb 9;14:1117052. doi: 10.3389/fphar.2023.1117052 (PMC9947157; doi:10.3389/fphar.2023.1117052)
Supplement: Supplementary file 3 [file DataSheet1.PDF]

Supplementary Material

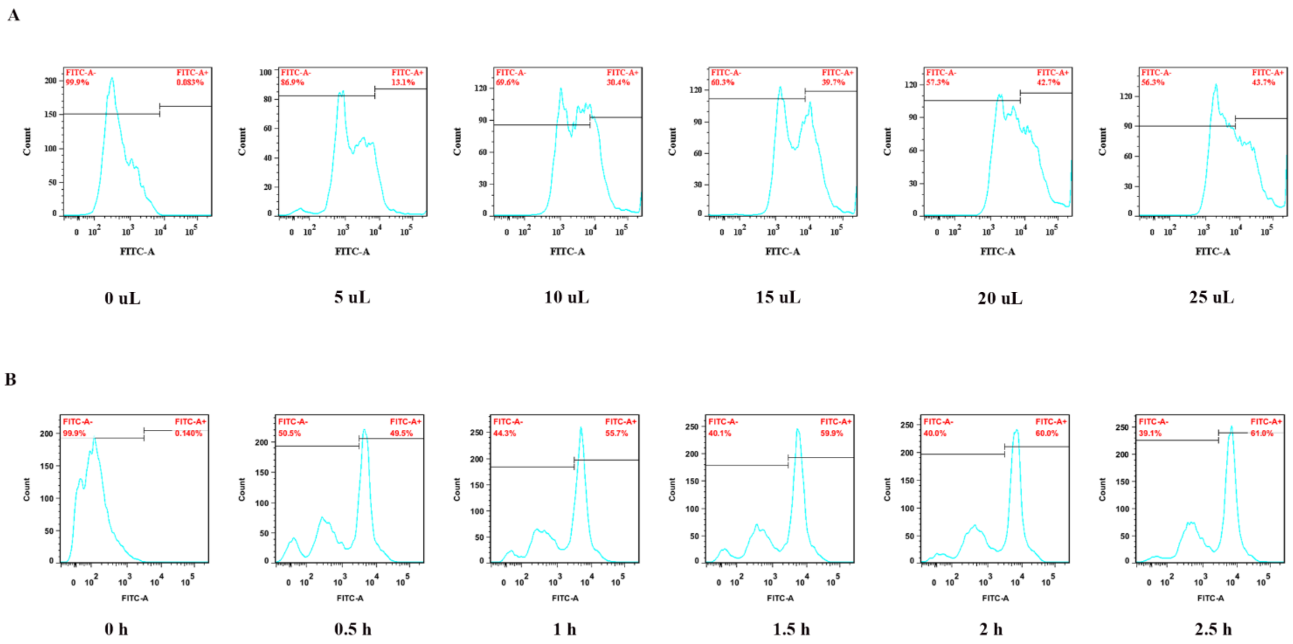

Figure S1. I Fluorescence intensities of FITC after different treatments. A and B, Different doses (**A**) and incubation times (**B**) of Atp-MSN (ICT@FITC) NPs.

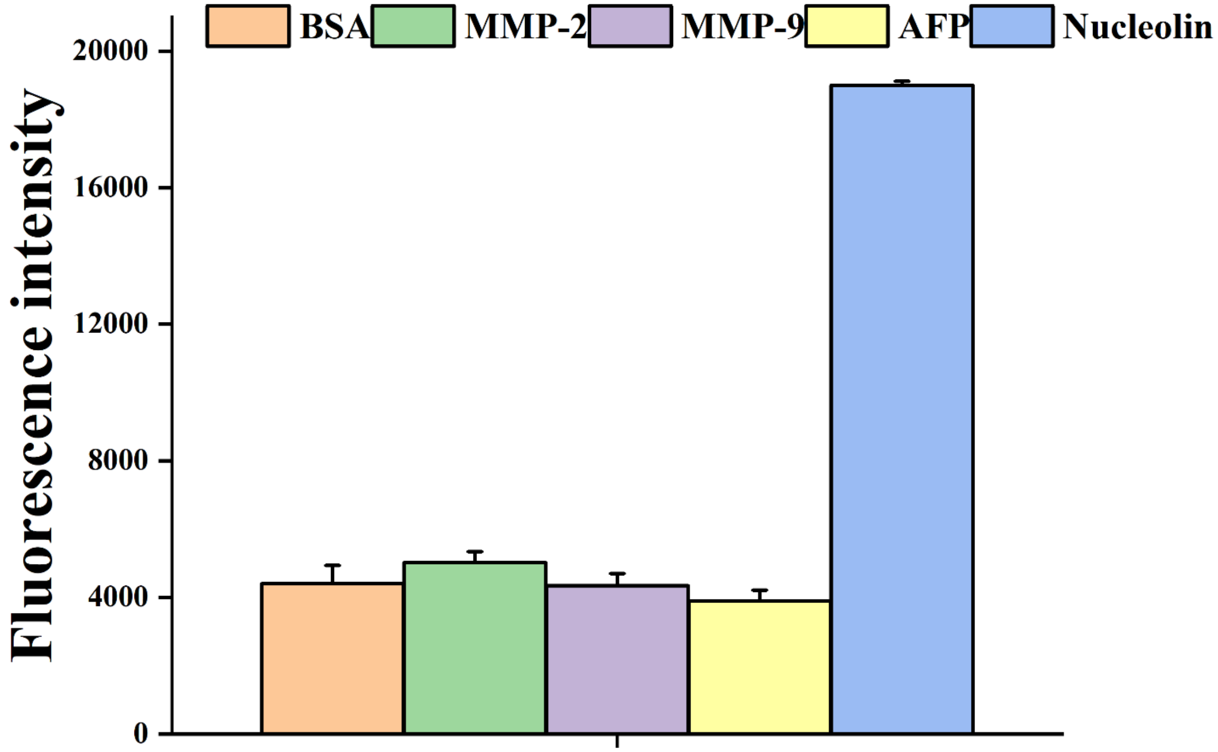

Figure S2. I Specific experiments. Fluorescence intensities of BSA, MMP-2, MMP-9, AFP and nucleolin after incubation with Atp-MSN (ICT@FITC) NPs.
